# Bis(1-methyl-1-phenyl­ethyl) peroxide

**DOI:** 10.1107/S1600536808033412

**Published:** 2008-10-18

**Authors:** Wei-Yi Su, Guang-Yang Hou, Qiu-Xiang Yin, Li-Na Zhou

**Affiliations:** a School of Chemical Engineering and Technology, Tianjin University, Tianjin 300072, People’s Republic of China

## Abstract

In the crystal structure, the title compound (also called dicumyl peroxide), C_18_H_22_O_2_, lies on a center of symmetry. The COOC plane including the di­oxy group makes a dihedral angle of 79.10 (5)° with the phenyl ring. An inter­molecular C—H⋯π inter­action is observed between the phenyl groups.

## Related literature

For general background, see: Ferrero (2006 ▶); Konar *et al.* (1993 ▶); Ramar & Alagar (2004 ▶); Wang *et al.* (1998 ▶).
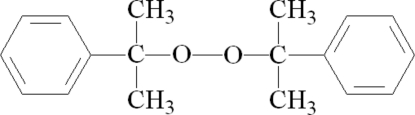


## Experimental

### 

#### Crystal data

C_18_H_22_O_2_
*M* *_r_* = 270.36
Orthorhombic, 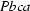

*a* = 10.040 (2) Å
*b* = 7.4774 (15) Å
*c* = 21.016 (4) Å
*V* = 1577.7 (5) Å^3^
*Z* = 4
Mo *K*α radiation
μ = 0.07 mm^−1^
*T* = 293 (2) K
0.25 × 0.20 × 0.15 mm

#### Data collection

Rigaku R-AXIS RAPID IP area-detector diffractometer
Absorption correction: multi-scan (**ABSCOR**; Higashi, 1995 ▶) *T*_min_ = 0.982, *T*_max_ = 0.989
11957 measured reflections
1464 independent reflections
1232 reflections with *I* > 2σ(*I*)
*R*_int_ = 0.077

#### Refinement

*R*[*F* ^2^ > 2σ(*F* ^2^)] = 0.043
*wR*(*F* ^2^) = 0.110
*S* = 1.05
1464 reflections
92 parameters
H-atom parameters constrained
Δρ_max_ = 0.26 e Å^−3^
Δρ_min_ = −0.18 e Å^−3^

### 

Data collection: *RAPID-AUTO* (Rigaku/MSC, 2004 ▶); cell refinement: *RAPID-AUTO*; data reduction: *CrystalStructure* (Rigaku/MSC, 2004 ▶); program(s) used to solve structure: *SHELXS97* (Sheldrick, 2008 ▶); program(s) used to refine structure: *SHELXL97* (Sheldrick, 2008 ▶); molecular graphics: *SHELXTL* (Sheldrick, 2008 ▶); software used to prepare material for publication: *SHELXTL*.

## Supplementary Material

Crystal structure: contains datablocks global, I. DOI: 10.1107/S1600536808033412/is2340sup1.cif

Structure factors: contains datablocks I. DOI: 10.1107/S1600536808033412/is2340Isup2.hkl

Additional supplementary materials:  crystallographic information; 3D view; checkCIF report

## Figures and Tables

**Table 1:** Hydrogen-bond geometry (Å, °) *Cg* is the centroid of the phenyl ring.

*D*—H⋯*A*	*D*—H	H⋯*A*	*D*⋯*A*	*D*—H⋯*A*
C3—H3*A*⋯*Cg*^i^	0.93	2.93	3.7874 (17)	154

Symmetry code: (i) 
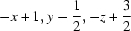
.

**Fig. 1.**
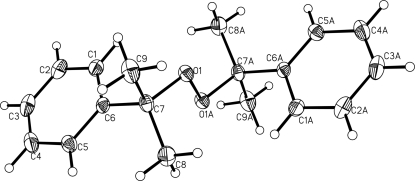
The molecular structure of the title compound, showing the atom-labelling scheme. Displacement ellipsoids are drawn at the 30% probability level.

**Fig. 2.**
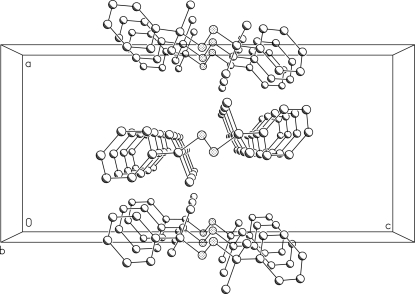
The packing diagram of (I), viewed down the b axis. H atoms have been omitted.

## supplementary crystallographic information

### Comment

The title compound, (I), a simple organic peroxide, has gradually become almost the most important additive in operations affected by molecular transport, such as grafting (Konar *et al.*, 1993; Ramar & Alagar, 2004) and cross-linking (Wang *et al.*, 1998; Ferrero, 2006), which are based on the formation of oxyradicals due to the thermal decomposition of the peroxides. It's widely used in the art as vulcanizing agents for resins and elastomers, as cross-linking agents for polyolefins.

The centrosymmetric molecular structure of (I) is shown in Fig. 1. In the molecule, two phenyl rings are, of course, parallel to each other due to the symmetry element. The peroxy unit has an O—O bond length of 1.6853 (16) Å, and the four atoms, C7, O1, O1A and C7A are coplanar with a C7—O1—O1A bond angle of 106.02 (9)°. There is no hydrogen bond in the packing structure, and cohesion of the crystal can be attributed to van der Waals interactions.

### Experimental

At room temperature, the title compound (1 g) provided by Gaoqiao petrochemical corporation was dissolved in 20 mL ethanol (99.7%). The solvent was vaporized slowly by use of a film covering the container (beaker). Then the solution was placed in darkness until crystals appeared. The product was taken out from the solvent by tweezers, and dried in the air at room temperature.

### Refinement

H atoms are placed in calculated positions and constrained to ride on their parent atoms, with C–H = 0.93–0.96 Å, and with *U*_iso_(H) = 1.2*U*_eq_(C) or 1.5*U*_eq_(methyl C).

### Crystal data

**Table:**

C_18_H_22_O_2_	*D*_x_ = 1.138 Mg m^−^^3^
*M**_r_* = 270.36	Melting point: 315.15 K
Orthorhombic, *P**b**c**a*	Mo *K*α radiation, λ = 0.71073 Å
Hall symbol: -P 2ac 2ab	Cell parameters from 8972 reflections
*a* = 10.040 (2) Å	θ = 3.5–27.6°
*b* = 7.4774 (15) Å	µ = 0.07 mm^−^^1^
*c* = 21.016 (4) Å	*T* = 293 K
*V* = 1577.7 (5) Å^3^	Plate, colorless
*Z* = 4	0.25 × 0.20 × 0.15 mm
*F*(000) = 584	

### Data collection

**Table:**

Rigaku R-AXIS RAPID IP area-detector diffractometer	1464 independent reflections
Radiation source: rotating anode	1232 reflections with *I* > 2σ(*I*)
graphite	*R*_int_ = 0.077
oscillation scans	θ_max_ = 25.5°, θ_min_ = 3.5°
Absorption correction: multi-scan (*ABSCOR*; Higashi, 1995)	*h* = −12→10
*T*_min_ = 0.982, *T*_max_ = 0.989	*k* = −9→9
11957 measured reflections	*l* = −25→25

### Refinement

**Table:**

Refinement on *F*^2^	Secondary atom site location: difference Fourier map
Least-squares matrix: full	Hydrogen site location: inferred from neighbouring sites
*R*[*F*^2^ > 2σ(*F*^2^)] = 0.043	H-atom parameters constrained
*wR*(*F*^2^) = 0.110	*w* = 1/[σ^2^(*F*_o_^2^) + (0.0556*P*)^2^ + 0.2734*P*] where *P* = (*F*_o_^2^ + 2*F*_c_^2^)/3
*S* = 1.05	(Δ/σ)_max_ = 0.010
1464 reflections	Δρ_max_ = 0.26 e Å^−^^3^
92 parameters	Δρ_min_ = −0.17 e Å^−^^3^
0 restraints	Extinction correction: SHELXL97 (Sheldrick, 2008), Fc^*^=kFc[1+0.001xFc^2^λ^3^/sin(2θ)]^-1/4^
Primary atom site location: structure-invariant direct methods	Extinction coefficient: 0.027 (7)

### Special details

**Table:**

Geometry. All e.s.d.'s (except the e.s.d. in the dihedral angle between two l.s. planes) are estimated using the full covariance matrix. The cell e.s.d.'s are taken into account individually in the estimation of e.s.d.'s in distances, angles and torsion angles; correlations between e.s.d.'s in cell parameters are only used when they are defined by crystal symmetry. An approximate (isotropic) treatment of cell e.s.d.'s is used for estimating e.s.d.'s involving l.s. planes.
Refinement. Refinement of *F*^2^ against ALL reflections. The weighted *R*-factor *wR* and goodness of fit *S* are based on *F*^2^, conventional *R*-factors *R* are based on *F*, with *F* set to zero for negative *F*^2^. The threshold expression of *F*^2^ > σ(*F*^2^) is used only for calculating *R*-factors(gt) *etc*. and is not relevant to the choice of reflections for refinement. *R*-factors based on *F*^2^ are statistically about twice as large as those based on *F*, and *R*- factors based on ALL data will be even larger.

### Fractional atomic coordinates and isotropic or equivalent isotropic displacement parameters (Å^2^)

**Table:**

	*x*	*y*	*z*	*U*_iso_*/*U*_eq_	
O1	0.46269 (8)	0.07789 (11)	0.51277 (4)	0.0309 (3)	
C1	0.44577 (13)	−0.10967 (18)	0.62787 (6)	0.0373 (4)	
H1A	0.3746	−0.1115	0.5996	0.045*	
C2	0.44768 (16)	−0.2285 (2)	0.67854 (6)	0.0462 (4)	
H2A	0.3785	−0.3099	0.6836	0.055*	
C3	0.55144 (16)	−0.2265 (2)	0.72135 (6)	0.0482 (4)	
H3A	0.5527	−0.3060	0.7554	0.058*	
C4	0.65322 (16)	−0.1055 (2)	0.71319 (6)	0.0453 (4)	
H4A	0.7231	−0.1025	0.7422	0.054*	
C5	0.65252 (13)	0.01215 (17)	0.66204 (6)	0.0353 (3)	
H5A	0.7227	0.0920	0.6568	0.042*	
C6	0.54838 (12)	0.01186 (16)	0.61873 (5)	0.0285 (3)	
C7	0.54171 (12)	0.14706 (16)	0.56434 (6)	0.0302 (3)	
C8	0.67730 (14)	0.20577 (19)	0.54013 (6)	0.0419 (4)	
H8A	0.7274	0.1027	0.5271	0.063*	
H8B	0.7242	0.2670	0.5734	0.063*	
H8C	0.6660	0.2847	0.5045	0.063*	
C9	0.45933 (16)	0.30860 (19)	0.58538 (6)	0.0457 (4)	
H9A	0.3740	0.2689	0.6004	0.068*	
H9B	0.4472	0.3880	0.5500	0.068*	
H9C	0.5051	0.3703	0.6190	0.068*	

### Atomic displacement parameters (Å^2^)

**Table:**

	*U*^11^	*U*^22^	*U*^33^	*U*^12^	*U*^13^	*U*^23^
O1	0.0353 (5)	0.0293 (5)	0.0282 (5)	0.0064 (4)	−0.0042 (4)	−0.0065 (3)
C1	0.0372 (7)	0.0434 (8)	0.0312 (7)	−0.0045 (6)	0.0036 (6)	0.0002 (6)
C2	0.0558 (9)	0.0452 (9)	0.0377 (8)	−0.0046 (7)	0.0142 (7)	0.0028 (6)
C3	0.0711 (11)	0.0426 (9)	0.0309 (7)	0.0163 (7)	0.0120 (7)	0.0058 (6)
C4	0.0556 (9)	0.0506 (9)	0.0297 (7)	0.0182 (7)	−0.0080 (7)	−0.0051 (6)
C5	0.0383 (7)	0.0358 (7)	0.0319 (7)	0.0045 (6)	−0.0042 (6)	−0.0074 (5)
C6	0.0330 (7)	0.0288 (7)	0.0235 (6)	0.0042 (5)	0.0017 (5)	−0.0057 (5)
C7	0.0377 (7)	0.0271 (6)	0.0258 (6)	−0.0014 (5)	−0.0039 (5)	−0.0034 (5)
C8	0.0495 (8)	0.0387 (8)	0.0376 (7)	−0.0153 (6)	−0.0015 (7)	0.0017 (6)
C9	0.0654 (10)	0.0339 (8)	0.0377 (8)	0.0124 (7)	−0.0072 (7)	−0.0068 (6)

### Geometric parameters (Å, °)

**Table:**

O1—C7	1.4392 (15)	C5—C6	1.3862 (17)
O1—O1^i^	1.4853 (16)	C5—H5A	0.9300
C1—C6	1.3871 (18)	C6—C7	1.5273 (17)
C1—C2	1.387 (2)	C7—C8	1.5181 (18)
C1—H1A	0.9300	C7—C9	1.5292 (18)
C2—C3	1.377 (2)	C8—H8A	0.9600
C2—H2A	0.9300	C8—H8B	0.9600
C3—C4	1.375 (2)	C8—H8C	0.9600
C3—H3A	0.9300	C9—H9A	0.9600
C4—C5	1.3893 (19)	C9—H9B	0.9600
C4—H4A	0.9300	C9—H9C	0.9600
			
C7—O1—O1^i^	106.02 (9)	O1—C7—C8	110.23 (10)
C6—C1—C2	121.05 (13)	O1—C7—C6	110.47 (10)
C6—C1—H1A	119.5	C8—C7—C6	113.76 (10)
C2—C1—H1A	119.5	O1—C7—C9	101.74 (10)
C3—C2—C1	120.35 (14)	C8—C7—C9	110.70 (11)
C3—C2—H2A	119.8	C6—C7—C9	109.28 (10)
C1—C2—H2A	119.8	C7—C8—H8A	109.5
C4—C3—C2	119.21 (14)	C7—C8—H8B	109.5
C4—C3—H3A	120.4	H8A—C8—H8B	109.5
C2—C3—H3A	120.4	C7—C8—H8C	109.5
C3—C4—C5	120.61 (13)	H8A—C8—H8C	109.5
C3—C4—H4A	119.7	H8B—C8—H8C	109.5
C5—C4—H4A	119.7	C7—C9—H9A	109.5
C6—C5—C4	120.72 (13)	C7—C9—H9B	109.5
C6—C5—H5A	119.6	H9A—C9—H9B	109.5
C4—C5—H5A	119.6	C7—C9—H9C	109.5
C5—C6—C1	118.05 (12)	H9A—C9—H9C	109.5
C5—C6—C7	121.56 (11)	H9B—C9—H9C	109.5
C1—C6—C7	120.31 (11)		
			
C6—C1—C2—C3	0.7 (2)	O1^i^—O1—C7—C6	−65.93 (12)
C1—C2—C3—C4	−0.1 (2)	O1^i^—O1—C7—C9	178.12 (10)
C2—C3—C4—C5	−0.7 (2)	C5—C6—C7—O1	155.72 (11)
C3—C4—C5—C6	0.95 (19)	C1—C6—C7—O1	−27.54 (15)
C4—C5—C6—C1	−0.32 (18)	C5—C6—C7—C8	31.13 (16)
C4—C5—C6—C7	176.48 (11)	C1—C6—C7—C8	−152.13 (12)
C2—C1—C6—C5	−0.51 (19)	C5—C6—C7—C9	−93.14 (14)
C2—C1—C6—C7	−177.36 (12)	C1—C6—C7—C9	83.59 (14)
O1^i^—O1—C7—C8	60.64 (13)		

Symmetry codes: (i) −*x*+1, −*y*, −*z*+1.

### Hydrogen-bond geometry (Å, °)

**Table:**

*D*—H···*A*	*D*—H	H···*A*	*D*···*A*	*D*—H···*A*
C3—H3A···Cg^ii^	0.93	2.93	3.7874 (17)	154

Symmetry codes: (ii) −*x*+1, *y*−1/2, −*z*+3/2.

## References

[bb1] Ferrero, F. (2006). *J. Therm. Anal. Calorim.* **83**, 373–378.

[bb2] Higashi, T. (1995). *ABSCOR* Rigaku Corporation, Tokyo, Japan.

[bb3] Konar, J., Sen, A. K. & Bhowmick, A. K. (1993). *J. Appl. Polym. Sci.* **48**, 1579–1585.

[bb4] Ramar, P. & Alagar, M. (2004). *Polym. Adv. Technol.* **15**, 377–381.

[bb5] Rigaku/MSC (2004). *RAPID-AUTO* and *CrystalStructure* Rigaku/MSC, The Woodlands, Texas, USA.

[bb6] Sheldrick, G. M. (2008). *Acta Cryst.* A**64**, 112–122. 10.1107/S0108767307043930 18156677

[bb7] Wang, Z., Chan, C.-M., Zhu, S.-H. & Shen, J.-R. (1998). *Polymer*, **39**, 6801–6806.

